# Radiation Resistance of the U(Al, Si)_3_ Alloy: Ion-Induced Disordering

**DOI:** 10.3390/ma11020228

**Published:** 2018-02-02

**Authors:** Louisa Meshi, Gili Yaniv, Pavel Horak, Jiri Vacik, Natalia Mykytenko, Gennady Rafailov, Itzchak Dahan, David Fuks, Arik Kiv

**Affiliations:** 1 Department of Materials Engineering, Ben-Gurion University of the Negev, P.O. Box 653, Beer-Sheva 8410501, Israel; gilusha1@gmail.com (G.Y.); fuks@bgu.ac.il (D.F.); kiv@bgu.ac.il (A.K.); 2 Nuclear Physics Institute, Academy of Sciences of the Czech Republic, Hlavni 130, Husinec-Rez 68250, Czech Republic; phorak@ujf.cas.cz (P.H.); vacik@ujf.cas.cz (J.V.); 3 South-Ukrainian National Pedagogical University after K. D. Ushinskij, Odessa 65000, Ukraine; mykytenkon@gmail.com; 4 Nuclear Research Center-Negev, P.O. Box 9001, Beer-Sheva 8419001, Israel; grafailov@gmail.com (G.R.); idahan60@gmail.com (I.D.)

**Keywords:** U-Al-Si, ion-irradiation, transmission electron microscopy, structural defects, disordering

## Abstract

During the exploitation of nuclear reactors, various U-Al based ternary intermetallides are formed in the fuel-cladding interaction layer. Structure and physical properties of these intermetallides determine the radiation resistance of cladding and, ultimately, the reliability and lifetime of the nuclear reactor. In current research, U(Al, Si)_3_ composition was studied as a potential constituent of an interaction layer. Phase content of the alloy of an interest was ordered U(Al, Si)_3_, structure of which was reported earlier, and pure Al (constituting less than 20 vol % of the alloy). This alloy was investigated prior and after the irradiation performed by Ar ions at 30 keV. The irradiation was performed on the transmission electron microscopy (TEM, JEOL, Japan) samples, characterized before and after the irradiation process. Irradiation induced disorder accompanied by stress relief. Furthermore, it was found that there is a dose threshold for disordering of the crystalline matter in the irradiated region. Irradiation at doses equal or higher than this threshold resulted in almost solely disordered phase. Using the program “Stopping and Range of Ions in Matter” (SRIM), the parameters of penetration of Ar ions into the irradiated samples were estimated. Based on these estimations, the dose threshold for ion-induced disordering of the studied material was assessed.

## 1. Introduction

In nuclear reactors, where U is used as a fuel, cladding is usually made of Al alloys (with Si as major alloying element). The fuel-cladding chemical interactions (both radiation driven or/and thermally assisted) are considered to be the life-limiting events of the fuel system. Thus, study of the U-Al-Si system, which is the main component of cladding-fuel interaction layer, is of crucial importance. The changes in the interaction layer under irradiation can lead to significant radiation effects in the fuel and to break the balance in the nuclear reactor as a whole. This layer can acquire a thermal conductivity that affects fuel performance [1,2], or it can become brittle such that, in the case of monolithic fuel, fuel/cladding separation could result [3]. Regarding the crystal structure of the U-alloys, it was reported that irradiation can cause partial or full amorphization of the U-Si and U-Mo/Al samples [4,5,6]. On the radiation resistance of U-Al-Si ternary aluminides which appear in the interaction layer little is known.

In general, U-Al-Si phase diagram was studied numerously. In [7] full phase diagram was constructed using thermodynamic modelling, based on experimental results. Quasi-binary UAl_3_-USi_3_ phase diagram is one of possible ways to present compositional range of the U-Al-Si system—relevant to the interaction layer. This quasi-binary phase diagram was and still is a matter of dispute. UAl_3_ and the USi_3_ phases are isostructural (both belonging to the cubic AuCu_3_ structure type). Given this fact it would be expected that their quasi-binary diagram should be isomorphous, with full substitution of Si and Al atoms at the “Cu” sub-lattice. However, previous studies have shown that this system does not display complete liquid and solid solubility, and conflicting results have been observed regarding the phases present in the system. Dwight reported complete solid solubility at 900 °C with indications that a miscibility gap exists at lower temperatures [8]. Weitzer et al., found the existence of a miscibility gap at 900 °C that causes a separation of U(Al, Si)_3_ solid solution into two phases, one rich in Al, U(Al_0.71_, Si_0.29_)_3_, and the other rich in Si, U(Si_0.6_, Al_0.4_)_3_ [9]. The existence of a miscibility gap was reported also by Nazare at 600 °C [10]. Other theoretical study [11] indicated complete solubility above 633 °C, and two ordered phases stable at low temperatures, each having a wide solubility range, that transform into a single ordered phase at 533 °C. In [12], experimental quasi-binary phase diagram was constructed exhibiting existence of one intermetallic phase at lower temperatures. Although in that research the crystal structure of this phase was not discussed, it was regarded as an ordered variant of the cubic U(Al, Si)_3_. It was shown that this phase is stable over a wide range of compositions (from 6 at % to 64 at % Si) [12]. Our group has solved the structure of this intermetallide [13,14]. The atomic structure of the ordered UAl_1.71_Si_1.29_ phase (corresponding to the U(Al_x_, Si_1−x_)_3_ stoichiometry with x = 0.57) was found to be tetragonal (I4/*mmm*, a = 8.347 Å and c = 16.808 Å) [13,14]. The behavior of this phase under the irradiation conditions was not checked previously and is a focus of current study.

In this work ion beams are used to simulate radiation effects under reactor irradiation in the indicated material [15]. One of the drawbacks of ion irradiation is the short penetration depth of ions and the continuously varying dose rate over the penetration depth. Therefore, for a correct comparison of radiation effects corresponding to different doses and energies, the regions at well-defined depths from the surface must be able to be sampled reproducibly. To meet this requirement the samples used in current research were of small thickness. Transmission electron microscopy (TEM) samples were irradiated directly. The same samples were characterized prior and after the irradiation by Ar ions fluxes of 10^13^, 10^14^, 10^15^, 10^16^, and 10^18^ ion/cm^2^.

Our results demonstrate that mosaic structure of the ordered phase has gradually disappeared as a function of the irradiation dose. Moreover, the domain free areas were less strained and exhibited disordered cubic U(Al, Si)_3_ structure. For interpretation of these results we used the models of ion implantation [16]. The program Stopping and Range of Ions in Matter (SRIM, http://www.srim.org/, USA) (v.2008) [17] was applied to estimate the parameters of the passage of ions in the material. Theoretical results are in agreement with the experimental data.

## 2. Materials and Methods

High purity uranium (99.99%), aluminum (99.999%), and silicon (99.9999%) were used to prepare U(Al_x_, Si_1−x_)_3_ alloy with x = 0.57 in arc melting furnace (VAR) under Ar atmosphere. To increase homogeneity, the sample was re-melted five times. Then, the alloy was heat treated at 800 °C for 336 h in an inert atmosphere of Ar.

The alloys were examined by powder X-ray diffraction (XRD), scanning electron microscopy (SEM, JEOL, Tokyo, Japan) and transmission electron microscopy (TEM). The local phase compositions were determined in SEM by energy-dispersive X-ray analysis (EDX) on polished un-etched cross-sections. For TEM sample preparation the disks were cut using a low-speed diamond saw. The disks were then mechanically polished on diamond paper to a thickness of 70 μm. Disks 3 mm in diameter were cut using a mechanical punch. Subsequently, thinning down to 30 μm was performed by a Gatan dimple grinder. Final thinning was done by a Gatan precision ion polishing system (PIPS) at 5 keV using an angle of maximum 6° to the surface of the samples. The specimens were examined at 200 kV using a Fas-TEM 2010 (JEOL, Japan) and JEM-2100F TEM (JEOL, Japan), both equipped with EDX analyzers (ThermoFisher Scientific, Waltham, MA, USA) and CCD cameras (Gatan, Pleasanton, CA, USA). The same samples were examined prior and after the irradiation.

The ion irradiation was performed using the Low Energy Ion System (LEIS) of the Nuclear Physics Institute (Prague, Czech Republic) which allows irradiating materials with ions in interval A = 1–200 with an energy in interval (100 eV–35 keV). A special sample holder was designed to irradiate samples by ions with necessary dose rate while maintaining the desired temperature. The tests showed that at ion currents of 100–400 µA the temperature can raise to hundreds of degrees. In our experiment the beam had to be focused, and in this state a hot spot of a millimeter size and a higher beam current is achieved. Therefore, a detailed analysis of the distribution of the ion density over the beam area was performed.

## 3. Results and Discussion

### 3.1. Experimental Results

#### 3.1.1. Initial Characterization of the Alloy

**Figure 1:**
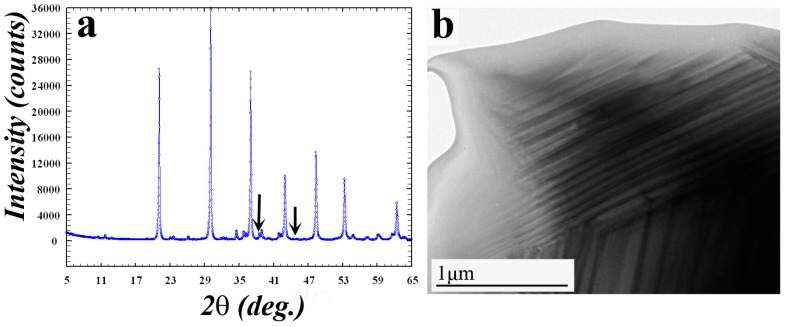
(**a**) X-ray powder diffraction pattern taken from the studied alloy. All peaks can be indexed in terms of ordered U(Al, Si)_3_ phase. Two peaks (marked by arrows) belong to pure Al. According to the low intensity of these peaks it can be understood that pure Al exists in low quantity in the alloy. (**b**) Typical bright field TEM image of a grain of the ordered U(Al, Si)_3_ phase. Domains, constituting this grain, are clearly seen.

**Figure 2:**
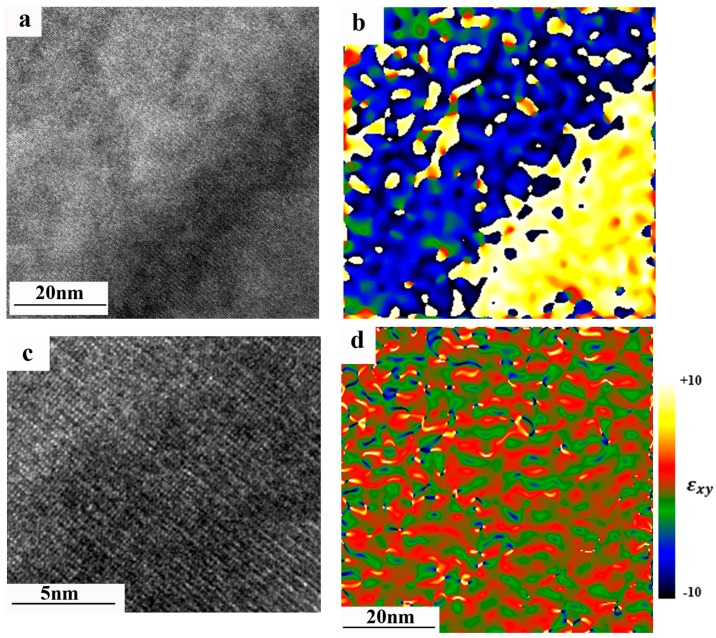
(**a**) HRTEM image of the interface between two domains of the ordered U(Al, Si)_3_ phase, taken at [110] orientation; (**b**) geometric phase map calculated from the HRTEM image using GPA. Differences in colors correspond to the phase change. Absolute values of the phase here are not important; (**c**) enlarged portion of the interface (darker part) shown in (a). Slight misorientation of the domains is seen; and (**d**) strain map. The legend of this map is shown on the right. It can be seen that green and red colors correspond to different signs of the strain (compression and tension).

Figure 1a shows the X-ray diffractogram taken from the studied alloy. It can be inferred that the alloy contains two phases: major ordered U(Al, Si)_3_ phase and minor Al. TEM analysis confirmed this conclusion. It should be specifically pointed out that disordered cubic U(Al, Si)_3_ phase was not found in the samples prior the irradiation. In addition, it was found that the grains of the ordered phases consisted of domains. Mosaicity of the U(Al, Si)_3_ grains was already reported in [14]. It was documented that heat treatment caused growth of the domain. In current research, even after the heat treatment, 10–30 nm wide domains still existed (see Figure 1b). Applying geometric phase analysis (GPA) [18] on the High Resolution Transmission Electron Microscope (HRTEM) images (Figure 2a), and the strain in the grains of ordered U(Al, Si)_3_ phase was assessed. For better visibility, enlarged image of the interface is shown in Figure 2c. Since GPA is based on phase retrieval, calculated in the Fourier (reciprocal) space, a geometric phase map (calculated directly from the HRTEM image (Figure 2a)) is shown in Figure 2b. Since this map shows variations from an ideal structure (chosen as reference), the displacement of the lattice fringes can be determined directly. Thus, this phase map illustrates the existence of domains boundaries. From the geometric phase map—strain map is constructed. On the constructed strain map (Figure 2d) it can be seen that domains are highly strained, containing both compression and tensile components, while the strain is uniformly distributed across the grain containing multiple domains. It should be noted that such evaluation of the strain at the domain boundaries was performed with great success for example on high-entropy AlCoCrFeNi alloy [19] proving the validity of this technique for such cases.

All TEM samples in the studied series were studied before the irradiation evaluating the approximate thickness, phase content and composition, so that it can be stated with high confidence that all pre-irradiated samples were similar.

#### 3.1.2. Samples after the Irradiation

**Figure 3:**
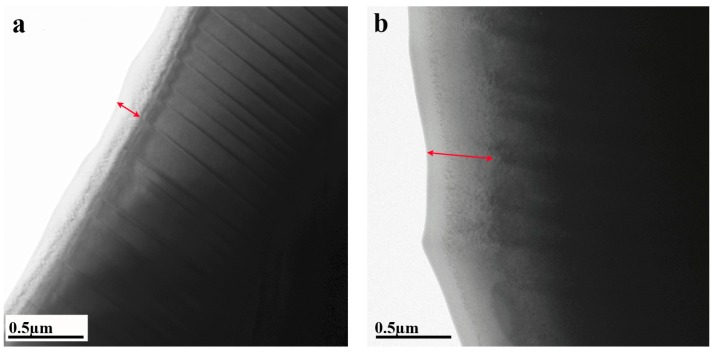
Bright field TEM images taken from the samples irradiated at doses (**a**) 10^13^ and (**b**) 10^15^ ions/cm^2^. The layer of disordered cubic U(Al, Si)_3_ phase (which appeared due to the irradiation) is marked by an arrow. It can be clearly seen that the layer becomes thicker when the dose is higher.

**Figure 4:**
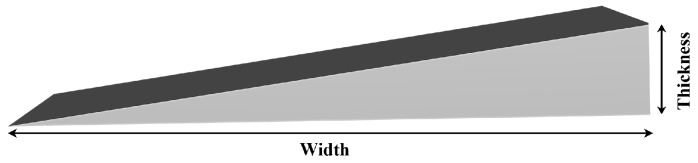
Scheme of half of the TEM sample in cross-section is shown (the area of minimal thickness is where hole starts). Terms “thickness” and “width” (as referred in current paper) are defined.

Same TEM samples, studied in previous section, were irradiated ex situ by Ar^+^ ions at 10^13^, 10^14^, 10^15^, 10^16^, and 10^18^ ion/cm^2^ fluxes. It should be noted that in order to not induce additional (to irradiation) effects, post-irradiated samples were neither ion milled nor plasma cleaned. Low magnification TEM images taken from samples exposed to doses 10^13^ and 10^15^ ion/cm^2^ are shown in Figure 3 as an example of the irradiation effect. It can be seen that near the hole of the TEM specimens (were the thickness of the sample is the smallest) layer of the disordered U(Al, Si)_3_ phase has formed. The width of the disordered U(Al, Si)_3_ layer has grown as the dose increases. For clarity, terms “thickness” and “width” (as referred in current paper) are defined on Figure 4, where scheme of TEM sample in cross section is shown. Table 1 lists the width of the disordered phase layer as a function of the irradiation flux. These results allowed estimation of the irradiation flux threshold of full order-disorder phase transition in the transparent to electrons thickness of the TEM specimen as 10^16^ ions/cm^2^, since 600 nm was the approximate width of most of the electron transparent areas. Moreover in the sample irradiated at 10^18^ ions/cm^2^ no ordered phase was found even at width more than 1 µm.

**Figure 5:**
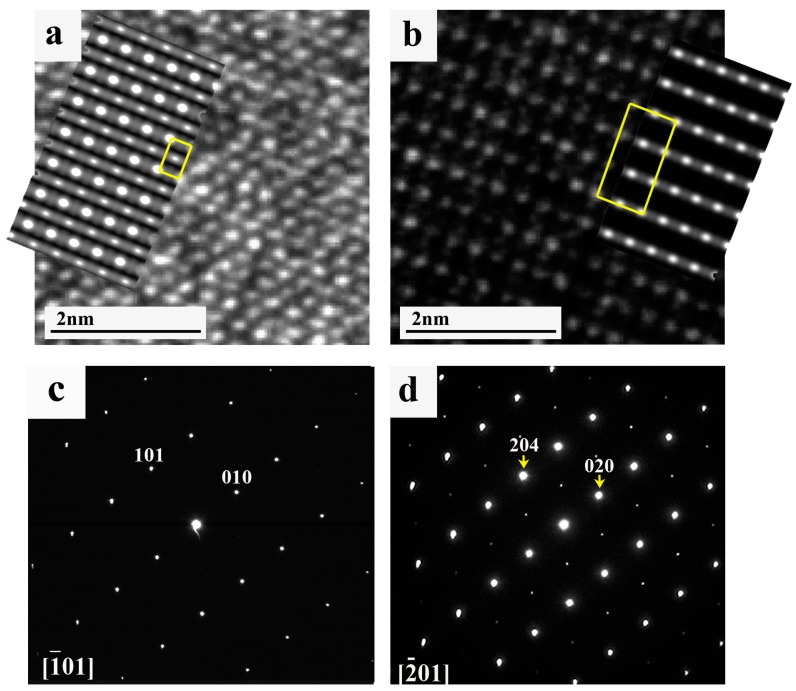
HRTEM images taken from the (**a**) disordered cubic and (**b**) ordered tetragonal U(Al, Si)_3_ phases at [$\bar{1}01$] and [$\bar{2}01$] orientations, respectively. Simulated images (shown in the insets) were prepared in JEMS [17] on the basis of the model presented in [9,10]. For clarity, the 2D unit cell is shown. (**c**,**d**) present corresponding electron diffraction patterns taken from these phases (i.e., disordered and ordered, respectively) at the same orientations.

Differentiation between the ordered and disordered U(Al, Si)_3_ phases is fairly easy. As shown in Figure 5, they differ both in the direct and in the reciprocal space. Simulation of the HRTEM images (prepared using JEMS software (version 4, Lausanne, Switzerland) [20]) is added in the insets and agreement between the experimental and simulated images is evident.

**Figure 6:**
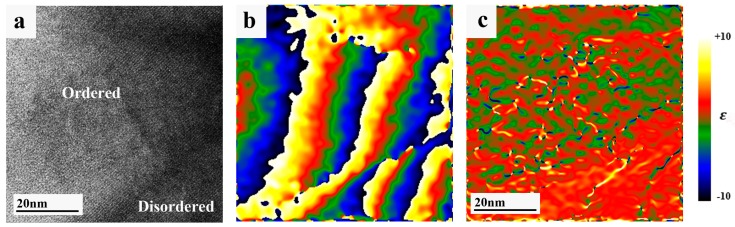
(**a**) HRTEM image of the interface between the disordered (due to irradiation) and ordered U(Al, Si)_3_ phases taken at [$\bar{1}01$] and [$\bar{2}01$] orientations, respectively; (**b**) geometric phase map calculated from the HRTEM image using GPA. Differences in colors correspond to the phase change. Absolute values of the phase here are not important; and (**c**) the strain map. The legend of this map is shown on the right. It can be seen that area in the right lower corner of the map is less strained with respect to the other areas, and it corresponds to the disordered phase.

Furthermore, in the disordered phase no reminiscence of the domains, which constituted the original ordered phase, was observed. Applying GPA on the HRTEM image taken at the ordered/disordered interface (Figure 6a)—strain was evaluated. The geometrical phase map shown in Figure 6b illustrates in a more clear way than the original HRTEM image the position of the boundaries of phases. Strain map (Figure 6c) observed at this area is completely different from the one shown in Figure 2c where only ordered phase was studied. As known, a creation of radiation defects removes mechanical stresses in materials [21], in particular, in the boundary regions of block structures. It should be pointed out that the values and the signs of the strains on the presented maps are relative, because GPA algorithm demands from user the definition of the reference position in the image where the strain is zero. Significant difference between Figure 2 and Figure 6 illustrates dramatic changes of strain as a function of irradiation dose.

### 3.2. Interpretation of the Results

It was found in [22] that in U-Al-Si type compounds the so-called heavy clusters are formed under ion irradiation. They occur due to the gathering of U atoms caused by their significant displacements from original positions. Such displacements are stimulated by the ion irradiation that knocks out the light atoms in the lattice. The growth of heavy clusters and the formation of a large amount of such clusters leads to the disordering of the U(Al, Si)_3_ lattice. The direct displacements of U atoms from their sites due to elastic collisions with the ions from the beam at the used ion energies are much less important [23]. Experimental results given in Section 3.1 indicate the existence of the threshold dose of the ion irradiation which is manifested by the damage of the domain structure in the as-received material.

To explain these data we consider the features of the energy losses of the irradiating ion beam penetrating into the material. It is known that while reducing the energy of bombarding ions, the energy losses caused by elastic collisions increase [24]. Respectively, the formation of structural defects caused by elastic collisions between the ions and the lattice atoms occurs efficiently for the ions having the energies at the end of the ion range. This means that the effective displacement of light atoms (i.e., Si, Al) should occur close to the stopping point of the implanted ions. This implies that the space distribution of the ion-induced structural defects in the irradiated region of the sample is similar to the space distribution of the implanted ions. Although these distributions do not coincide, and, real distributions in both cases deviate from the Gaussian one, we can use Equation (1) [25] which determines the distribution of concentration of implanted ions *n*(*x*) to estimate the ratio of concentrations of structural defects at different depths inside the crystal:

(1) $$n\left(x\right)=\frac{F}{\sqrt{2\pi }\Delta R_{p}}exp\left[-\frac{{\left(x-R_{p}\right)}^{2}}{2\Delta R_{p}^{2}}\right]$$

where *R_p_* is a projected range of ions, Δ*R_p_* is a standard deviation, or the spread of distribution of the concentration of implanted ions in the vicinity of *R_p_*, and *F* is the ion irradiation dose.

**Figure 7:**
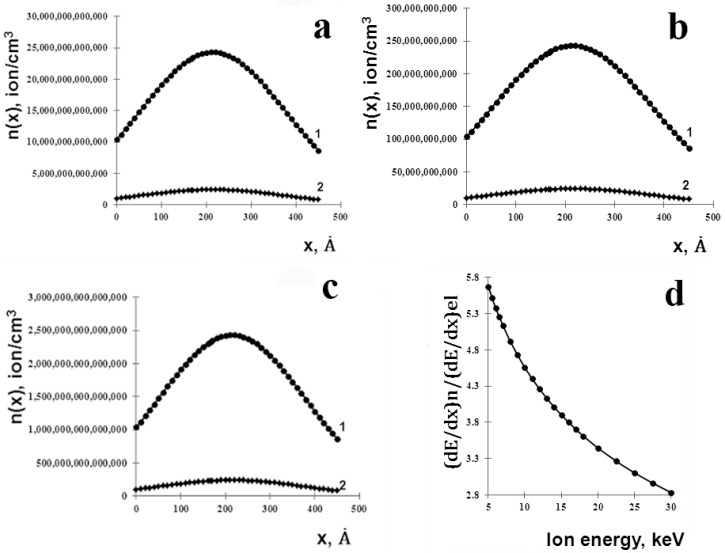
The depth distribution of the Ar ions from the surface of irradiated material (*x*) for the total doses of (**a**) 10^14^ (1) and 10^13^ (2) ion/cm^2^; (**b**) 10^16^ (1) and 10^15^ (2) ion/cm^2^; and (**c**) for the total doses of 10^18^ (1) and 10^17^ (2) ion/cm^2^; and (**d**) The relationship between the elastic (*dE/dx*)*_n_* and inelastic (*dE/dx*)*_el_* losses as a function of ion energy.

The parameters in Equation (1) were calculated using the program SRIM 2008 [17,26]. The description of physical models and algorithms used in this software can be found in [26]. For Ar^+^ penetration into the U(Al, Si)_3_ compound the following input parameters were used: energy of Ar^+^ ions, *E* = 30 keV, atomic number of Ar^+^ in periodic table, Z = 18, and its atomic mass A = 39.9. The density of the ordered U(Al, Si)_3_ compound was calculated as 7.5 g/cm^3^ using the crystallographic data listed in [13,14]. As a result, *R_p_* = 178 Å and Δ*R_p_* = 103 Å were obtained, and the calculated (Equation (1)) spatial distributions of implanted Ar^+^ ions in investigated compound are shown in Figure 7a–c).

Since elastic collisions dominate near the stopping point of bombarding ions, it can be assumed that formation of structural defects in the vicinity of the given coordinate *x* is produced by ions with concentration *n*(*x*) (Figure 7a–c). This value can be numerically equated to the ion irradiation dose (*F*_1_), which induced structural damage at the point *x*, because, in fact, the number of ions which have passed over the irradiation time through an area of 1 cm^2^ is not less than the number of ions per 1 cm^3^ that were accumulated at the point *x* during the exposure time.

It is seen from Figure 7a–c that the concentration of Ar^+^ ions at the surface is ~2.5 times less than at the depth *R_p_*. Approximately the same ratio can be taken for the concentration of structural defects. To estimate the concentration of structural defects at point x connected with the displacement of light atoms (as mentioned above), it is necessary to know the concentration of light atoms in the material and the cross section for their displacement. Using unit cell parameters of the ordered U(Al, Si)_3_ phase [13,14], the concentration of light atoms in this material was calculated as *N*_0_ = 4.1 × 10^22^ cm^−3^.

The typical cross-section for displacement of such atoms due to elastic collisions is *σ_d_* ≈ 3 − 5 × 10^−16^ cm^2^ [27]. Concentration of displaced light atoms, *N_d_*, may be calculated with Equation (2) [24]:

(2) $$N_{d}=F_{1}\sigma_{d}N_{0}\nu$$

where *σ_d_* is the cross-section for the displacement of light atoms due to elastic collisions, *ν* = *E*/2*E_d_* is the multiplication factor of displaced atoms (or the number of displaced atoms per one primary knocked atom (PKA) [28]), *E* is an energy of PKA, and *E_d_* is the energy that should be transferred to the lattice atom for its displacement from the lattice site.

Using Figure 7a–c we estimated the concentration of structural defects under the appropriate ion irradiation doses that were created on the average at point x in the area from the surface to the depth *R_p_*. Resultant *F*_1_ values for different irradiation doses are presented in Table 2.

To assess the magnitude of *ν*, it is necessary to know the energy of ions, predominantly involved in formation of structural defects, i.e., the ions whose energy is mostly consumed for elastic collisions. For this purpose using SRIM program the calculation of energy losses due to elastic collisions, $-{\left(\frac{dE}{dx}\right)}_{n}$, and due to electronic excitations, $-{\left(\frac{dE}{dx}\right)}_{el}$, for a number of energy values was performed. The calculations were carried out for the energies of Ar^+^ ions in the interval (0–30 keV), and the ratio of these magnitudes inside this interval is presented in Figure 7d. It may be concluded from this figure that for *E* = 8 keV this ratio is approximately equal to 5. In this case ~80% of the energy of the ions is spent on elastic collisions. Due to the fact that the masses of Ar^+^ ions and the light atoms in the crystal (Al and Si) are close, the energy of PKA is also ~8 keV. Taking *E_d_* ≈ 20 eV [29] the magnitude for *ν* is ≈ 0.2 × 10^3^.

Applying Equation (2) total dose was calculated as *N_d_* ≈ 3.3 × 10^22^ cm^−3^ in the case of the dose of implanted ions 10^16^ ion/cm^2^, meaning that, approximately every second atom from the sub-lattice of light atoms (Al, Si) will be knocked out from its lattice site. Thus, it is clear that for the ion dose 10^16^ ion/cm^2^ we can expect a significant disordering of this sublattice. An increase in the irradiation dose of ions (10^18^ ion/cm^2^) did not change the observed disordering effect. These conclusions are in line with the results listed in previous section.

## 4. Summary

Ion-induced disordering of the U(Al, Si)_3_ compound, potential constituent of the fuel-cladding interaction layer in the nuclear reactor, was studied. TEM samples with an approximate thickness (in the electron transparent area) of 0–100 nm were irradiated by Ar ions at 30 keV. TEM investigation was performed prior and after the irradiation. Before the ion irradiation only an ordered U(Al, Si)_3_ phase with mosaic grains (constituted of domains) was found. After the irradiation, ions have induced disorder and, as a function of the ion dose, the amount of the disordered phase grew at the expense of the ordered one. These results can be correlated to the literature, where other related alloys were studied following irradiation. Although in [4,5,6] amorphization occurred, in our case studied intermetallides underwent disordering and it can be assumed that higher dosages would have induced amorphization.

The U(Al, Si)_3_ grains stopped exhibiting the domains at the dose 10^16^ ion/cm^2^, resulting in almost solely disordered phase. The ion implantation models were used to explain the existence of the ion dose threshold in the ion-induced disordering process. Analysis of the constructed strain maps demonstrate that, as the result of ion irradiation, the initial strain, accumulated at the domains’ boundaries, significantly decreased.

## Figures and Tables

**Table 1:** Width of the disordered phase layer which appears after the irradiation and respective irradiation doses. Since the width of the layers varied, the error of measurement is not shown, while it should be noted that we assess the uncertainty of the measurement in this case to be no more than 10 nm.

Dose $\left(\frac{\mathrm{ion}}{{\mathrm{cm}}^{2}}\right)$	10^16^	10^15^	10^14^	10^13^
Width (nm)	600	400	220	150

**Table 2:** Estimated values of parameter *F*_1_ as a function of irradiation dose.

Dose $\left(\frac{\mathrm{ion}}{{\mathrm{cm}}^{2}}\right)$	10^18^	10^17^	10^16^	10^15^	10^14^	10^13^
*F*_1_ $\left(\frac{\mathrm{ion}}{{\mathrm{cm}}^{2}}\right)$	1.5 × 10^15^	1.5 × 10^14^	1.5 × 10^13^	1.5 × 10^12^	1.5 × 10^11^	1.5 × 10^10^
